# Supplementary material for: The C. elegans gonadal sheath Sh1 cells extend asymmetrically over a differentiating germ cell population in the proliferative zone
Source: eLife. 2022 Sep 12;11:e75497. doi: 10.7554/eLife.75497 (PMC9467509; doi:10.7554/eLife.75497)
Supplement: Transparent reporting form [file elife-75497-transrepform1.docx]

***eLife’s* transparent reporting form**

We encourage authors to provide detailed information *within their submission* to facilitate the interpretation and replication of experiments. Authors can upload supporting documentation to indicate the use of appropriate reporting guidelines for health-related research (see EQUATOR Network), life science research (see the BioSharing Information Resource), or the ARRIVE guidelines for reporting work involving animal research. Where applicable, authors should refer to any relevant reporting standards documents in this form.

If you have any questions, please consult our Journal Policies and/or contact us:

editorial@elifesciences.org.

# Sample-size estimation

- You should state whether an appropriate sample size was computed when the study was being designed
- You should state the statistical method of sample size computation and any required assumptions
- If no explicit power analysis was used, you should describe how you decided what sample (replicate) size (number) to use

Please outline where this information can be found within the submission (e.g., sections or figure legends), or explain why this information doesn’t apply to your submission:

We did not perform *a priori* sample size estimates. The question of statistical power is most relevant for the experiments shown in Figure 1, which measures positions of the Sh1 cells (measured repeatedly in the rest of the paper) and the germ cells transition in nuclear morphology (which was previously published by Cinquin et al. (2010) and Fox and Schedl (2015). Our results agreed with previously measured values for both the Sh1 and germ cell landmarks. Most importantly, the permissive temperature controls showed highly significant differences between the germ cell and Sh1 landmarks at permissive temperature, and between the germ cell landmarks at permissive and restrictive temperatures. Therefore, the lack of difference between the Sh1 and gc landmarks at the restrictive temperature can be considered to be a real lack of difference.

# Replicates

- You should report how often each experiment was performed
- You should include a definition of biological versus technical replication
- The data obtained should be provided and sufficient information should be provided to indicate the number of independent biological and/or technical replicates
- If you encountered any outliers, you should describe how these were handled
- Criteria for exclusion/inclusion of data should be clearly stated
- High-throughput sequence data should be uploaded before submission, with a private link for reviewers provided (these are available from both GEO and ArrayExpress)

Please outline where this information can be found within the submission (e.g., sections or figure legends), or explain why this information doesn’t apply to your submission:

Morphologically abnormal gonads (for which gonad elongation does not result in a distal tip at the dorsal midbody) were excluded from analysis of Sh1 position. These made up ~20% of DG5020 and DG5131 animals examined. These numbers are given in the relevant Results sections. For brood size assays, all progeny of animals that were lost (killed during transfer or burrowed into the dish) were discarded from analysis. Biological replicates only are reported in the paper. Technical replicates were performed during investigator training (two investigators counted the same plate for brood size, for example).

# Statistical reporting

- Statistical analysis methods should be described and justified
- Raw data should be presented in figures whenever informative to do so (typically when N per group is less than 10)
- For each experiment, you should identify the statistical tests used, exact values of N, definitions of center, methods of multiple test correction, and dispersion and precision measures (e.g., mean, median, SD, SEM, confidence intervals; and, for the major substantive results, a measure of effect size (e.g., Pearson's r, Cohen's d)
- Report exact p-values wherever possible alongside the summary statistics and 95% confidence intervals. These should be reported for all key questions and not only when the p-value is less than 0.05.

Please outline where this information can be found within the submission (e.g., sections or figure legends), or explain why this information doesn’t apply to your submission:

We used ANOVA with Tukey’s multiple comparison tests to assess significance of the difference in mean position for Sh1 fluorescence signal and germ cell nuclear morphology transition. These tests are described in every figure legend, and the software used is described in the Methods.

 (For large datasets, or papers with a very large number of statistical tests, you may upload a single table file with tests, Ns, etc., with reference to sections in the manuscript.)

# Group allocation

- Indicate how samples were allocated into experimental groups (in the case of clinical studies, please specify allocation to treatment method); if randomization was used, please also state if restricted randomization was applied
- Indicate if masking was used during group allocation, data collection and/or data analysis

Please outline where this information can be found within the submission (e.g., sections or figure legends), or explain why this information doesn’t apply to your submission:

The brood size assay was masked from the investigators doing the counting; they knew strain names but not genotypes.

# Additional data files (“source data”)

- We encourage you to upload relevant additional data files, such as numerical data that are represented as a graph in a figure, or as a summary table
- Where provided, these should be in the most useful format, and they can be uploaded as “Source data” files linked to a main figure or table
- Include model definition files including the full list of parameters used
- Include code used for data analysis (e.g., R, MatLab)
- Avoid stating that data files are “available upon request”

Please indicate the figures or tables for which source data files have been provided:

Source data files with raw measurements are uploaded.
